# Joint Analysis of Radiative and Non-Radiative Electronic Relaxation Upon X-ray Irradiation of Transition Metal Aqueous Solutions

**DOI:** 10.1038/srep24659

**Published:** 2016-04-21

**Authors:** Ronny Golnak, Sergey I. Bokarev, Robert Seidel, Jie Xiao, Gilbert Grell, Kaan Atak, Isaak Unger, Stephan Thürmer, Saadullah G. Aziz, Oliver Kühn, Bernd Winter, Emad F. Aziz

**Affiliations:** 1 Institute of Methods for Material Development, Helmholtz-Zentrum Berlin, Albert-Einstein-Straße 15, D-12489 Berlin, Germany; 2 Department of Chemistry, Freie Universität Berlin, Takustraße 3, D-14159 Berlin, Germany; 3 Institut für Physik, Universität Rostock, D-18051 Rostock, Germany; 4 Department of Chemistry, Graduate School of Science, Kyoto University, Kitashirakawa-Oiwakecho, Sakyo-Ku, Kyoto 606-8502, Japan; 5 Chemistry Department, Faculty of Science, King Abdulaziz University, 21589 Jeddah, Saudi Arabia; 6 Department of Physics, Freie Universität Berlin, Arnimallee 14, D-14159 Berlin, Germany

## Abstract

L-edge soft X-ray spectroscopy has been proven to be a powerful tool to unravel the peculiarities of electronic structure of transition metal compounds in solution. However, the X-ray absorption spectrum is often probed in the total or partial fluorescence yield modes, what leads to inherent distortions with respect to the true transmission spectrum. In the present work, we combine photon- and electron-yield experimental techniques with multi-reference first principles calculations. Exemplified for the prototypical FeCl_2_ aqueous solution we demonstrate that the partial yield arising from the Fe3s → 2p relaxation is a more reliable probe of the absorption spectrum than the Fe3d → 2p one. For the bonding-relevant 3d → 2p channel we further provide the basis for the joint analysis of resonant photoelectron and inelastic X-ray scattering spectra. Establishing the common energy reference allows to assign both spectra using the complementary information provided through electron-out and photon-out events.

X-ray spectroscopic methods are indispensable for probing the structure of matter. The recent developments in the vacuum liquid-microjet technique have broadened the applicability of especially soft X-ray spectroscopy to highly volatile liquid solutions^1^,^2^. The microjet in conjunction with X-rays provides *in situ* access to the electronic structure of atomic and molecular solutes. Hence, solute–solvent interactions as well as short-lived chemically reactive intermediates produced upon X-ray excitation or ionization can be studied in great detail^1^,^2^,^3^,^4^. Among the prominent applications is the investigation of aqueous transition-metal (TM) ions, in particular iron-water complexes, which play a key role in biological and physical-chemical processes^5^,^6^,^7^,^8^,^9^,^10^,^11^.

In L-edge soft X-ray spectroscopy of TM ions, the excitation and ionization of 2p core electrons gives insight into chemical bonding and functionality due to the dipole-allowed probing of frontier d-orbitals. Upon absorption of a soft X-ray photon, a 2p core hole is created at the metal centre; this photon energy is large enough to ionize an electron from the oxygen 1s orbital of the water solvent. Subsequent radiative decay channels of the core-excited TM ion comprise 3d → 2p and 3s → 2p pathways which represent about 1% of the total decay probability for the first-row TM ions^12^. The majority channel is due to Auger-electron decay; other autoionization processes although being possible^13^,^14^,^15^ will not be considered here.

In pioneering studies^16^,^17^,^18^,^19^,^20^,^21^, L-edge X-ray absorption (XA) spectra from aqueous TM ions have been reported, based, however, solely on the detection of the total fluorescence yield (TFY). The TFY detection mode corresponds to an action spectroscopy. It enjoys popularity since the direct measurement of XA in a transmission experiment is challenging for a liquid-jet with a typical diameter of 10–20 μm^1^,^22^,^23^,^24^,^25^. In a TFY measurement, a photodiode is used that integrates the signal from all possible radiative decay channels including the solvent background. However, individual relaxation channels have not been explored separately, with the 3d → 2p one being an exception^19^,^26^. In general, the assumption that the detected TFY signal intensity is proportional to the number of initially absorbed X-ray photons at a given excitation photon energy is not valid^27^,^28^. There are X-ray optical effects responsible for the deviation between TFY and true XA signals such as concentration-dependent self-absorption and saturation as well as signal/background ratio variation^29^,^30^. On a more fundamental level, in order to resemble the first-order XA spectrum the corresponding second-order fluorescence must follow an incoherent two-step model^31^, and the resulting signal is a product of absorption and emission spectra. This holds true only when the interference effects between different radiative decay channels and non-trivial dependence on the polarization of incoming and detected photons can be neglected. This assumption was found to be violated in several studies^27^,^28^. Moreover, the state-dependent electron delocalization into the neighbouring ligands after core-excitation could influence the fluorescence intensity^19^,^20^,^32^. Such charge migration processes may occur on a timescale comparable to the few femtosecond lifetime of the core hole^33^. These effects together, and our lack of understanding of the role of various decay channels, have caused a fair amount of speculations about the electronic structure which is often calculated by semi-empirical theoretical models^34^.

Disentangling individual channels requires to resolve the energy of the emitted second-order electrons and photons. This is possible through resonant photoelectron (RPE) spectroscopy for the electron channels, and via resonant inelastic X-ray scattering (RIXS) spectroscopy for photon-decay channels. In the present study, RIXS and RPE experimental techniques are applied together with the first principles restricted active space self-consistent field/second order perturbation theory (RASSCF/RASPT2) calculations in order to explore the complementary character of photon- and electron-emission spectra for the exemplary case of an FeCl_2_ aqueous solution. The focus is put on the interplay of the decay channels, which is addressed by detecting state-dependent pathways involving 3d valence orbitals versus stronger-bound 3p and 3s iron orbitals. The joint analysis of experimental and theoretical spectra provides two key results. First, the question is answered under which conditions partial-yield photon and electron detection can give an accurate measurement of the true X-ray absorption from TM aqueous solutions. As mentioned before, a transmission spectrum cannot be measured with the present cylindrical microjet, but this has no effect on the analysis of the multiple relaxation channels which is the focus of the present work. Partial fluorescence yield (PFY) and partial electron yield (PEY) spectra are obtained by integration of a signal within photon and electron channel-dependent energy ranges. Second, the basis for a back-to-back analysis of RPE and RIXS measurements from the same FeCl_2_ aqueous solution is established, providing the assignment of the decay channels and energies of the respective electronic states. It is shown that the joint interpretation of RIXS and RPE spectra for the 3d → 2p channel can considerably contribute to our understanding of solute–solvent interactions.

## Results and Discussion

**Figure 1:**
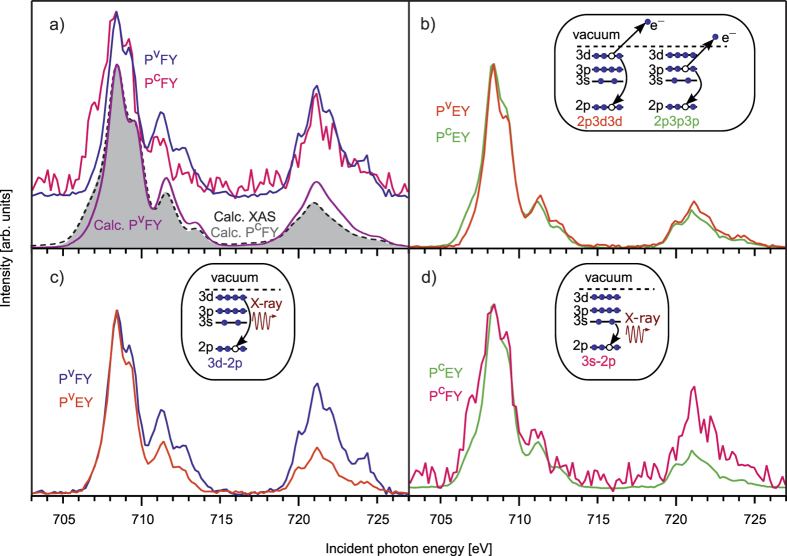
X-ray absorption spectra recorded in PEY and PFY modes for different decay channels. Comparison of (**a**) experimental valence and core PFY versus theoretical XAS and PFY spectra; (**b**) valence and core PEY; (**c**) valence spectra involving 3d core-hole refill; (**d**) core spectra due to 3p (P^c^EY) and 3s (P^c^FY) relaxations. The corresponding relaxation channels are depicted as insets. All spectra are normalized to the intensity of the highest peak at 708.4 eV to ease comparison.

The XA spectra from the iron aqueous solutions obtained in different partial-yield modes all exhibit L_3_ (706–715 eV) and L_2_ (719–726 eV) edge signals arising from the spin-orbit coupling of the 2p core-hole created by X-ray excitation. Figure 1 summarizes the PFY and PEY spectra, obtained for the various decay channels as indicated in the insets. The assignment of the different Auger-electron channels is presented in Figure S1 in the Supplementary Information (SI). The specific relaxation channels differ with respect to the refill of the core-hole which can be either from valence- or core-shells. For photon detection, the signal from Fe 3d → 2p and 3s → 2p relaxation channels was integrated over the emitted photon energy within the channel-dependent energy ranges 660–720 eV and 605–640 eV, respectively. Analogously, photoelectron spectra from 2p3d3d and 2p3p3p Auger decay channels were integrated within 672–715 eV and 560–600 eV kinetic energy ranges, resulting in the respective PEY-XA spectra.

The different decay channels can be classified according to the nature of the involved orbitals. In PFY (Fe 3d → 2p) and PEY (2p3d3d) the electron, which refills the core hole comes from a Fe 3d valence orbital (spectra are denoted as P^v^FY and P^v^EY). These 3d orbitals carry information on the mixing with ligand-centred orbitals. In contrast, in PFY (Fe 3s → 2p) and PEY (2p3p3p) the core-hole is refilled by electrons from deeper (3s/3p) Fe orbitals which do not appreciably interact with the ligands (spectra are denoted as P^c^FY and P^c^EY).

The calculated P^v^FY, P^c^FY spectra, and the transmission XA spectrum shown in Fig. 1a will serve as reference for the following discussion. The two PFY spectra exhibit distinct differences, i.e. the pre-edge intensity near 707 eV excitation photon energy is lower, while the post-edge at 709–714 eV and the L_2_ edge are more intense for P^v^FY than for P^c^FY. The calculated XA and P^c^FY spectra are essentially indistinguishable from each other. Further, both computed P^v^FY and P^c^FY spectra are in very good agreement with the experimental results that are also shown in Fig. 1a. Notice, however, that the calculations did not take into account the X-ray optical effects mentioned above.

The difference in the L_3_/L_2_ edge intensity ratio observed in Fig. 1 can be attributed to several effects. These include self-absorption as well as Coster-Kronig transitions, both enhancing the photon emission at the L_2_ edge^4^. The fact that these effects are not included into the calculation points to yet another reason for the difference in the spectra, i.e., the spin-orbit coupling. For the two radiative relaxation channels (3s → 2p and 3d → 2p) core-excited intermediate states are mixtures of the wave functions with different multiplicities due to the strong 2p core-hole spin-orbit coupling, whereas valence initial and final states almost do not mix, and represent pure quintet and triplet states. Thus, the second-order fluorescence process, initial → intermediate → final state, can be described as quintet (ground) → mixed → quintet and triplet states, for details see ref. 35. This leads to the appearance of new formally spin-forbidden radiative channels for L_3_ post-edge and L_2_ bands, where spin-orbit coupling is stronger than for the lower energy wing of the L_3_-edge^20^,^26^,^35^. Since spin-orbit coupling has no effect for 3s orbitals and is notable for 3d orbitals, it leads to distortions of P^v^FY as compared to the XA spectrum, whereas P^c^FY is not affected. In the following discussion we will focus on the L_3_ edge only, including its post-edge region.

Compared to the fluorescence yield mode, the P^v^EY and P^c^EY spectra (Fig. 1b) show similar although less pronounced differences; see also panels c and d of Fig. 1. The analysis of spin-orbit coupling is more involved in case of PEY, due to transitions between mixed intermediate states of the Fe^2+^ ion and final valence spin manifolds of the ionized Fe^3+^ system. Hence, these effects are not considered here.

Another source of differences between PEY/PFY spectra with respect to the transmission XA spectrum is the electronic interaction of the TM ion with the environment, i.e., solvation-shell water molecules. The excited electron can be further involved in other relaxation processes such as electron delocalization and energy transfer^20^,^36^. The delocalized excited electron has a lower probability to refill the 2p hole within its lifetime (sub-10 fs), and hence the indirect PEY/PFY probe should be state-dependent according to the extent of delocalization and should not track the true X-ray absorption probability^20^. The observed behaviour in Fig. 1 thus correlates with the orbital extensions: core 3s and 3p orbitals are strongly localized, and are not or barely involved in iron–ligand orbital mixing. Thus core orbitals are subject to less distortion since delocalization and also spin-orbit channels are of minor importance, while both channels are essential for the 3d shell, since 3d and water lone-pair orbitals are considerably mixed. This behaviour seems to be generally valid, for another example see refs 19,20.

The preceding discussion has pointed to the different sensitivity of valence- and core-level relaxation channels to electron delocalization and spin-orbit coupling. Hence, it is not surprising that P^c^EY and P^c^FY detection (Fig. 1c) leads to spectra which are more closely reflecting the true X-ray absorption. Here we argue that the computed XA spectrum accurately captures the true (transmission) XA spectrum; this is yet to be confirmed experimentally though.

**Figure 2:**
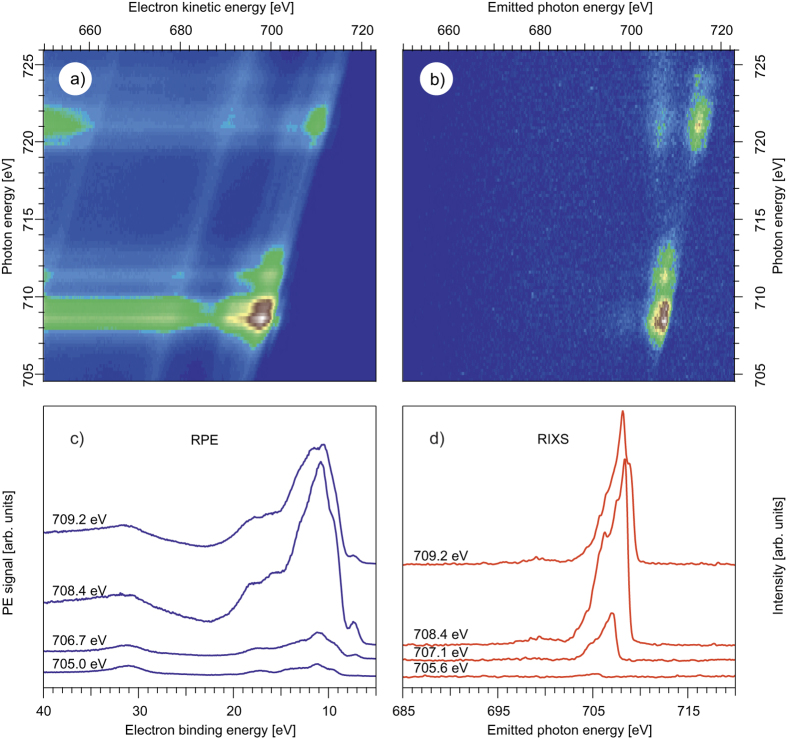
RPE and RIXS due to Fe 3d → 2p relaxation channel. (**a**) 2D RPE as a function of electron kinetic energies; (**b**) 2D RIXS; (**c**) 1D RPE cuts for the selected excitation energies, the electron binding energy is used to facilitate comparison with the RIXS spectra; (**d**) 1D RIXS cuts for the selected excitation energies.

In the following we focus on the particular electron- or photon-energy resolved channels involving frontier Fe 3d orbitals. The RPE and RIXS spectra measured at the Fe 2p → 3d resonance are shown in Fig. 2a,b, respectively, in a two-dimensional (2D) representation depending on the incoming photon energy. Panels c and d of Fig. 2 display four cuts through the 2D spectra in the L_3_-edge region at those excitation energies which correspond to the main absorption features (compare Fig. 1). The theoretical direct photoionization signal from the iron centre contributing to total RPE is shown in Figure S2 of the SI, and the calculated RIXS spectrum is very similar to that discussed in ref. 26.

**Figure 3:**
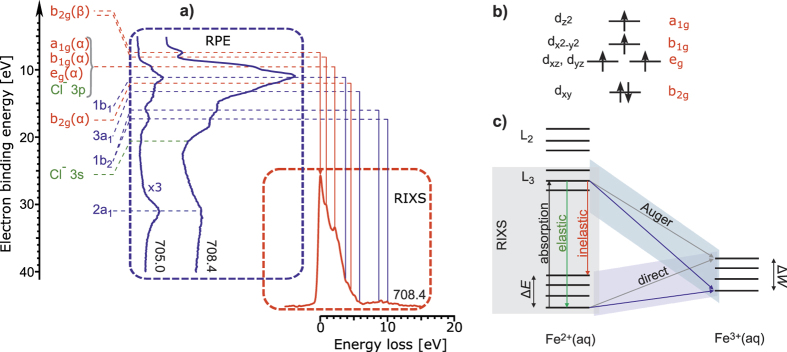
Assignment of RPE and RIXS. (**a**) Aligned RPE and RIXS spectra. Red, blue, and green labels correspond to Fe, H_2_O, and Cl^−^ transitions, respectively; (**b**) Sketch of Fe 3d orbital symmetries for weakly Jahn-Teller distorted system; (**c**) Scheme connecting the corresponding transitions in RIXS (elastic, inelastic) and RPE (both direct and Auger ionization channels).

The RPE spectrum is assigned with the help of the well-understood off-resonant photoelectron spectrum, obtained here at 705 eV excitation energy (see Figs 2c and 3a). It essentially represents the neat-water PE spectrum^37^,^38^ with the features at 11.2, 13.5, 17.3, and 31.0 eV binding energies (BE) being due to ionization from the 1b_1_, 3a_1_, 1b_2_, and 2a_1_ water valence orbitals (blue labels in Fig. 3a), respectively. A very weak signal from Fe 3d at 7.09 eV^39^ corresponding to the lowest ionization energy of the solution can be also seen.

Since the energetic structure of the valence levels does not depend on the excitation energy, we can discuss the principle features of the RPE spectra for an excitation energy for which signal intensities are strongest. This is the case at maximum absorption, *i.e.*, at 708.4 eV, shown in Fig. 3a. The large Auger-electron signal in the ~8–20 eV BE range, occurring for the Fe L_3_-edge excitation, mainly arises from the 2p3d3d Auger transition. The Fe 3d orbitals of weakly the tetragonally distorted Jahn-Teller d^6^ system with approximate D_4h_ point group symmetry split into four components as shown in Fig. 3b. According to the theoretical photoelectron spectrum (Figure S2 of the SI), the Fe transitions (red labels in Fig. 3a) group into three bands: a distinct feature due to ionization of a spin-down (β) electron from the lowest b_2g_(β) orbital at 7.09 eV BE, a broad band comprising numerous main and combination transitions of spin-up (α) electrons from a_1g_(α), b_1g_(α), and e_g_(α) orbitals overlapping in energy and being centred at 9.5 eV BE, and from b_2g_(α) at 12.24 eV BE. The b_2g_(β) electron occupying the deepest 3d orbital has the lowest ionization energy because the orbital binding energy is compensated by pairing (exchange) energy. The chloride 3p valence ionization (green labels in Fig. 3a) leads to a small contribution around 9 eV BE^40^ coinciding with the Fe a_1g_(α)/b_1g_(α)/e_g_(α) feature as well as with the low-binding energy side of the water 1b_1_ orbital signal. The feature at 15.3 eV is most probably due to first-solvation shell water 3a_1_ orbitals, which form σ-bonding combinations with iron a_1g_ and b_1g_ (d_z^2^_ and d_x^2^_−_y^2^_) orbitals. Note that the 1b_2_ water signal is also shifted to higher binding energies upon such interactions.

For the resonant excitation at 708.4 eV, the overall RPE signal intensity dramatically increases in comparison to the off-resonant region. Moreover, a signal increase occurs at the energies of the water peaks 1b_1_, 3a_1_, and 1b_2_, and of the Fe 3d peaks. Such a strong signal enhancement results from the interference of the outgoing electron waves from two simultaneous electron emission processes sharing the same final state (see Fig. 3c), and leading to identical kinetic energies of the emitted electrons^41^,^42^. One process is the direct ionization of the valence orbitals excited by the 708.4 eV energy photon. The other process is the 2p3d3d Auger-decay releasing a 3d electron from the valence level with the same kinetic energy. This interference effect is a qualitative unequivocal evidence of the mixing between iron and water valence orbitals, and represents a central experimental finding of this study. Note that if there was no mixing, only the Fe 3d signal would be enhanced.

A quantitative comparison of RPE and RIXS spectra in terms of the energies of the occurring peaks requires a proper alignment of both spectra on the energy axis. As a consequence of strong correlation effects and pronounced spin-mixing this requires switching from the one-electron orbital picture to the many-electron states picture as illustrated in Fig. 3c. The lowest-energy peak in the RPE spectrum is due to the transition between ground states of initial Fe^2+^(aq) and ionized Fe^3+^(aq) systems for direct ionization or between the X-ray core-excited state of Fe^2+^(aq) and the ground state of Fe^3+^(aq) as shown by blue arrows in Fig. 3c. This corresponds to elastic scattering in RIXS when the same core-excited state relaxes back to the ground state of Fe^2+^(aq) (green arrow in Fig. 3c).

The alignment of the lowest-binding energy RPE feature with the elastic RIXS peak, as is done in Fig. 3a, establishes a common energy reference which allows connecting all other features of the two spectra. Let us consider a particular valence excited state. In RPE, it corresponds to the transition to a final valence excited state of Fe^3+^(aq) with the excitation energy Δ*W* (cf. grey arrows in Fig. 3c). Analogously, the inelastic RIXS transition (red arrow) results in a valence excited state of Fe^2+^(aq) with excitation energy Δ*E*. If electronic relaxation upon ionization Fe^2+^(aq) → Fe^3+^(aq) + e^−^ is small and Δ*E* ≈ Δ*W*, the present alignment allows the back-to-back comparison and assignment of RPE and RIXS.

According to the suggested alignment, the feature at 2.13 eV loss energy in RIXS is connected with the broad band at 9.5 eV BE in RPE, and thus can be assigned to predominant radiative relaxation from a_1g_, b_1g_, and e_g_ orbitals. The RIXS spectral shoulder near 5.13 eV which is in accordance with the 12.24 eV shoulder in RPE can be attributed to the relaxation from the b_2g_ orbital, and a weak band near 9.0 eV loss energy is assigned to weak charge-transfer transitions from first-solvation shell water orbitals. The charge-transfer transitions again evidence the orbital mixing between metal and ligand. Remarkably, the 0.9 eV RIXS feature arises from the relaxation of the same b_2g_(β) electron as in the first RPE peak. The energy shift of 0.9 eV can be explained taking into account that in RPE the b_2g_(β) electron is completely removed from the system, and its corresponding Coulomb and exchange energies contribute to the ionization energy. In contrast, in RIXS the energy of the final state is determined by the change of the corresponding terms due to excitation of this electron from b_2g_ to other orbitals. The assignment based on our spectra alignment is supported by the theoretical analysis of the direct photoionization and the RIXS spectra.

Since RPE and RIXS transitions have different selection rules, having a common reference in energy does not imply that the intensities are the same. This explains the absence of prominent RIXS transitions involving water orbitals. Thus, the intensity differences provide information on the rate of different radiative and non-radiative transitions following the core-excitation.

An assignment of the higher-energy excitations, a particularly near 712 eV, where the PEY and PFY spectra exhibit substantially different intensities (Fig. 1), cannot be made based on our spectra. According to the theoretical analysis, this region corresponds to strong mixing between states of different multiplicities, and thus is difficult to interpret taking into account the different influence of spin-orbit coupling onto electron- and photon-out events.

## Conclusions

Complementary electron and photon emission spectra are necessary for a full understanding of electronic-structure interactions between metal solute and water solvent. Previously reported X-ray spectroscopy studies relied on the photon detection and did not consider the electronic structure information from the electron-emission channels. The parallel consideration of PFY and PEY spectra, originating from various channels, reveals large deviations from the true XA spectrum in the case of the 3d → 2p relaxation. In contrast, the “core” PFY and PEY spectra due to the 3s → 2p relaxation are accurate measures of the transmission XA spectrum. In fact our measurements suggest that the latter channel (investigated here for fluorescence) is the most accurate probe. Looking at the 3d → 2p relaxation, with the discussed energy alignment, one can find experimentally find the correspondence between spectral signatures in the RPE and RIXS spectra. Moreover, comparing RPE and RIXS intensities one may obtain information on the competition between the radiative and non-radiative decay channels. Our analysis suggests a new protocol for investigation of X-ray spectra of transition metal solute–solvent electronic interactions. The provided insight shall promote understanding structure and rational design of catalytic and other functional materials.

## Methods

### Experimental

FeCl_2_ hexahydrate salt, purchased from Sigma Aldrich with >99% purity, was dissolved in deionised water to make a 1 M aqueous solution for both photon and electron measurements. The colour of the solution is pale-lime and the molar ratio of [Fe(H_2_O)_5_Cl]^+^ and [Fe(H_2_O)_6_]^2+^ species is ~77/23 43,44. The microjet technique was employed to introduce the liquid sample into the vacuum chamber through a 20 μm diameter glass nozzle. The electron and photon signals were taken from the laminar area of the jet which is approximately 2–3 mm in length starting from the nozzle head.

The experiments were carried out at the U41-PGM undulator beamline at the synchrotron facility BESSY II, Berlin. Two experimental stations, LiXEdrom and LiquidPES, were used for photon and electron detection, respectively. The U41-PGM beamline provides horizontally linear polarized light with high photon flux and a micro-focus spot, which is crucial to micro-jet measurements. In LiXEdrom, photons emitted from the jet were collected along the polarization direction of the incident photon beam (to suppress the elastic peak) and subsequently dispersed by a spherical grating with 1200 lines/mm and 7.5 m radius. The dispersed photons were then detected by a microchannel plate (MCP)/fluorescence screen/CCD assembly. The sample, grating, and photon detector are arranged on a Rowland circle geometry for accurate focusing. For the LiquidPES experiment, electrons were collected perpendicular to the polarization of the incident photon beam by a hemispherical energy analyzer. Note that the 2p3s3s Auger-decay channel is not considered because of a too low signal. The details of the two experimental stations have been described elsewhere^1^,^2^,^3^,^4^.

### Computational

Theoretical calculations were performed for [Fe(H_2_O)_6_]^2+^ and [Fe(H_2_O)_5_Cl]^+^ complexes, where geometries were optimized at the BLYP/cc-pVTZ level with Gaussian 09 package^45^. The valence- and core-excited electronic states were further studied using the restricted active space self-consistent field (RASSCF) method^46^ with the relativistic ANO-RCC basis sets^47^,^48^ of triple-zeta quality. The second order perturbation theory correction (RASPT2) with an imaginary level shift of 0.1 Hartree was applied on top of RASSCF to account for dynamic correlation. The active space for XAS, RIXS, and PFY calculations included 2p in the RAS1 space (1 hole allowed) and 3s together with 3d orbitals in RAS2 space (full CI); all occupied inactive orbitals were kept frozen. The resulting configuration space includes dipole-allowed 2p → 3d excitations and 3d → 2p and 3s → 2p radiative relaxation transitions. Spin-orbit coupling was treated in the *LS*-coupling limit^49^ taking into account 75 quintet (*S* = 2) and 375 triplet (*S* = 1) directly interacting states. Scalar relativistic effects were included within the Douglas-Kroll-Hess approach^50^,^51^. RIXS and PFY spectra were evaluated using the Kramers-Heisenberg expression properly including coherence effects and polarization dependence (for details, see ref. 35). Valence photoelectron spectra were obtained using the Dyson orbital approach as described in ref. 52 with the active space including only 3d orbitals in RAS2 space. A single quintet initial state for [Fe(H_2_O)_6_]^2+^ and 1 sextet (*S* = 5/2), 24 quartet (*S* = 3/2), and 75 doublet (*S* = 1/2) final states coupled via spin-orbit coupling were included for [Fe(H_2_O)_6_]^3+^. All calculations were performed with a locally modified MOLCAS 8.0 code^53^.

## Additional Information

**How to cite this article**: Golnak, R. *et al*. Joint Analysis of Radiative and Non-Radiative Electronic Relaxation Upon X-ray Irradiation of Transition Metal Aqueous Solutions. *Sci. Rep.* **6**, 24659; doi: 10.1038/srep24659 (2016).

## Supplementary Material

Supplementary Information
