# Supplementary material for: Joint Analysis of Radiative and Non-Radiative Electronic Relaxation Upon X-ray Irradiation of Transition Metal Aqueous Solutions
Source: Sci Rep. 2016 Apr 21;6:24659. doi: 10.1038/srep24659 (PMC4838826; doi:10.1038/srep24659)
Supplement: Supplementary Information [file srep24659-s1.pdf]

Supporting Information

Joint Analysis of Radiative and Non-Radiative Electronic Relaxation Upon X-ray Irradiation of Transition Metal Aqueous Solutions

Ronny Golnak,[1,2] Sergey I. Bokarev,[3*] Robert Seidel,[1] Jie Xiao,[1] Gilbert Grell,[3] Kaan Atak,[1] Isaak Unger,[1] Stephan Thürmer,[4] Saadullah G. Aziz,[5] Oliver Kühn,[3] Bernd Winter,[1*] and Emad F. Aziz[1,6*]

[1]*Institute of Methods for Material Development, Helmholtz Zentrum Berlin, Albert-Einstein-Strasse 15, D-12489 Berlin, Germany*

[2]*Department of Chemistry, Freie Universität Berlin, Takustrasse 3, D-14159 Berlin, Germany*

[3]*Institut für Physik, Universität Rostock, D-18051 Rostock, Germany*

[4]*Department of Chemistry, Graduate School of Science, Kyoto University, Kitashirakawa-Oiwakecho, Sakyo-Ku, Kyoto 606-8502, Japan*

[5]*Chemistry Department, Faculty of Science, King Abdulaziz University, 21589 Jeddah, Saudi Arabia*

[6]*Department of Physics, Freie Universität Berlin, Arnimallee 14, D-14159 Berlin, Germany*

**Corresponding authors:*

sergey.bokarev@uni-rostock.de (S.I.B.), bernd.winter@helmholtz-berlin.de (B.W.), emad.aziz@helmholtz-berlin.de (E.F.A.)

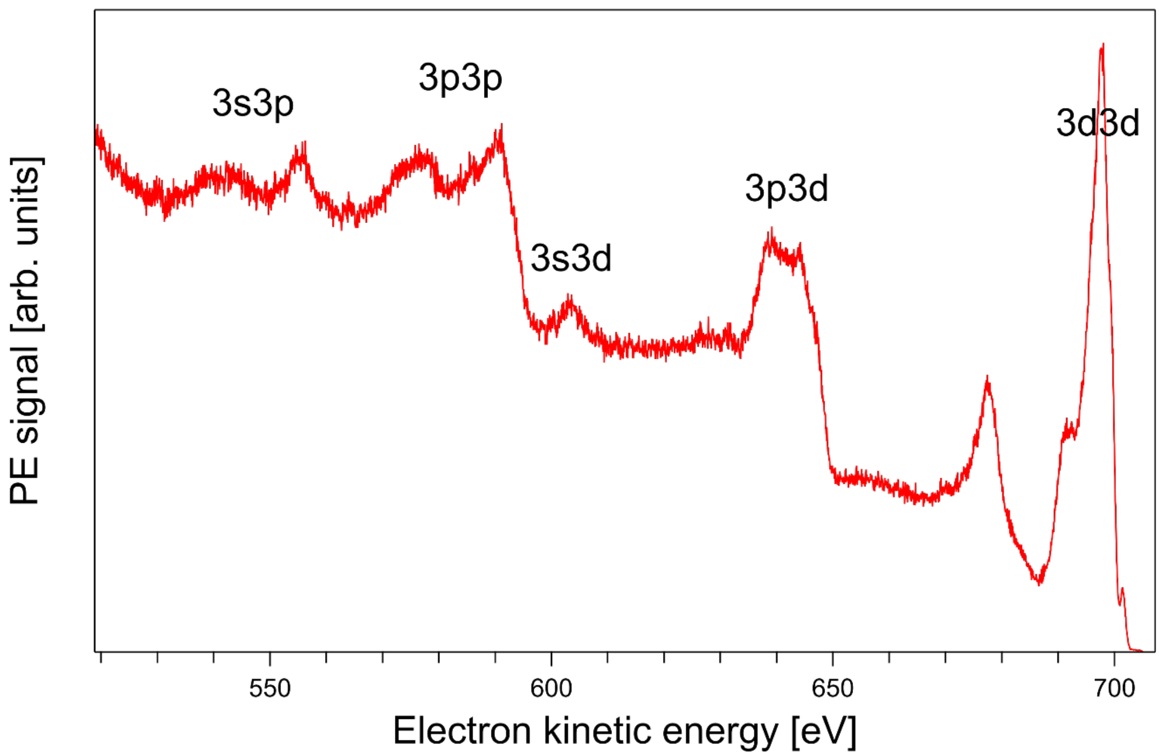

Figure S1. Full-range iron Auger-electron spectrum from 0.25 M $FeCl_2$ aqueous solution obtained at the L_3-edge, at 708.4 eV photon energy. Different Auger channels are labeled.

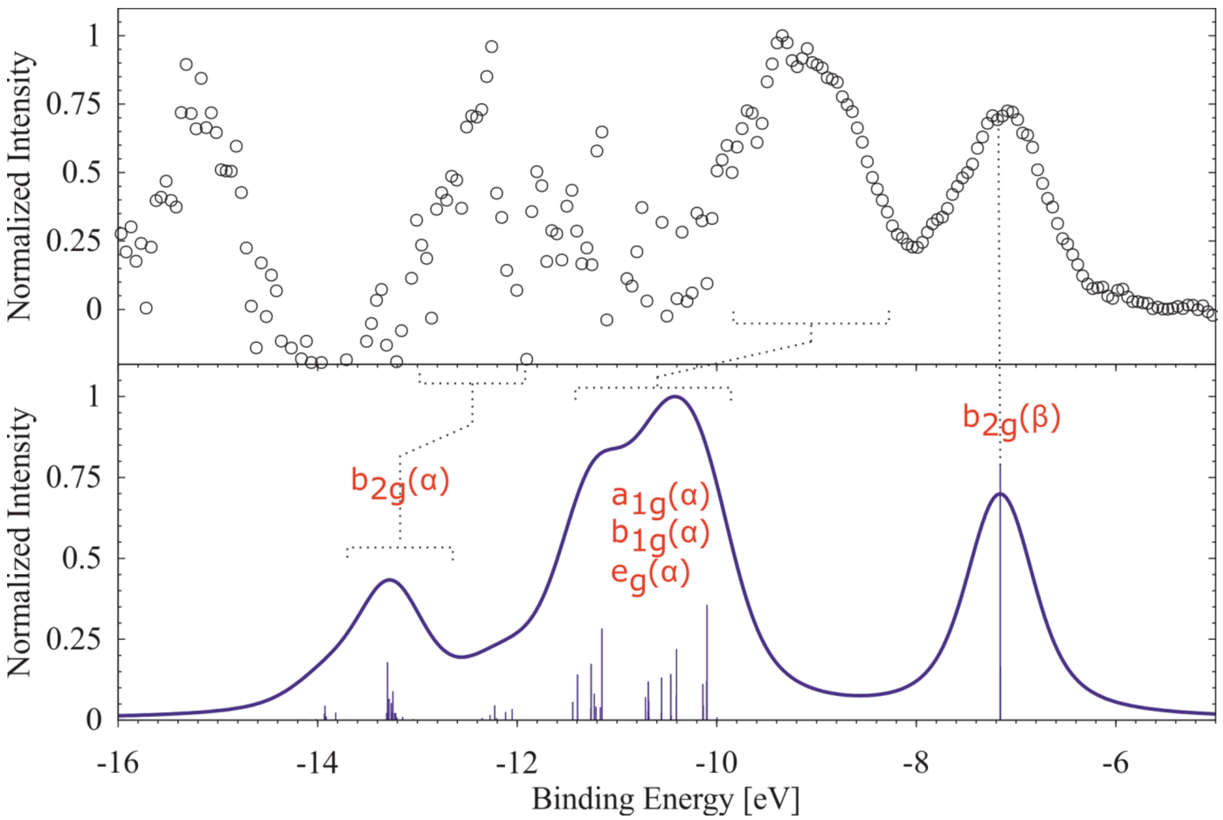

Figure S2. Comparison of the experimental difference spectrum (0.25 M $FeCl_2$ solution – 0.5 M NaCl solution) recorded at 200 eV incoming photon energy with (top figure) the calculated photoelectron spectrum of $[Fe(H_2O)_6]^{2+}$ (bottom). For orbital notation see Figure 3b of the main text.
